# Evaluation of the analgesic potential of serratus block associated with tap block in female dogs undergoing mastectomy

**DOI:** 10.29374/2527-2179.bjvm000925

**Published:** 2025-09-04

**Authors:** Isabele de Matos Oliveira, Desirée Santos da Rosa, Laryssa Reginaldo Ribeiro da Silva, Yasmin Santos Kaulich de Souza, Mateus Siarense Ribeiro, Gustavo Nunes de Santana Castro

**Affiliations:** 1 Universidade Iguaçu, c I, Nova Iguaçu, RJ, Brazil

**Keywords:** pain management, regional anesthesia, canine, controle da dor, anestesia regional, caninos

## Abstract

Breast tumors are common in female dogs, and in most cases, unilateral mastectomy is the surgical technique. This procedure poses a considerable risk for the development of post-surgical chronic pain. Therefore, the animal must have a good analgesic plan during the intraoperative period. This study aimed to evaluate the analgesic potential of the serratus plane (SP) block when combined with the transversus abdominis plane (TAP) block during the intraoperative and postoperative periods in female dogs undergoing mastectomy. Eighteen female dogs, aged 5–15 years and weighing 4–77 lbs, with mammary tumors staged I–III, were referred for total unilateral mastectomy. These dogs were pre-medicated with 0.03 mg/kg intramuscular acepromazine, an anesthetic was induced with propofol, and maintained with isoflurane. The dogs were divided into two groups: in 10 dogs, TAP blocks were performed with a volume of 0.4 mL/kg, combined with SP blocks with a volume of 0.4 mL/kg of 0.25% bupivacaine intraoperatively, before starting surgery, and in another eight dogs, tumescent anesthesia was performed with a cold solution containing lactated Ringer's, 2% lidocaine without vasoconstrictor, and adrenaline in 15 mL/kg intraoperatively. During data collection, analysis, interpretation of results, and subject follow-up, no group exhibited a response to nociceptive stimuli during the intraoperative period. In the postoperative period, only one dog from group 1 presented with a score greater than 5, and a slight delay in anesthetic recovery was observed in dogs from group 2. The evaluation of parameters during and after surgery, combined with the low need for analgesics in most female dogs, indicated that the combination of TAP and SP blocks was effective for analgesic control. In conclusion, this combination is an alternative to promote analgesia and favor recovery in female dogs undergoing mastectomy.

## Resumo

Tumores mamários são muito comuns em cadelas e, na maioria dos casos, a técnica cirúrgica adotada é a mastectomia unilateral. Este procedimento apresenta um risco considerável para o desenvolvimento de dor crônica pós-cirúrgica, então é de grande importância para o animal ter um bom plano analgésico durante o período intraoperatório. Este estudo tem como objetivo avaliar o potencial analgésico do bloqueio serrátil associado ao bloqueio do plano transverso do abdômen (TAP block) no período intraoperatório e pós-operatório de cadelas submetidas a mastectomia. Dezoito cadelas, com idades entre 5 e 15 anos, pesando de 4 a 77 libras, com tumores mamários classificados como estágios I a III, foram encaminhadas para mastectomia unilateral total. Essas cadelas foram pré-medicadas com acepromazina intramuscular na dose de 0,03 mg/kg, a indução anestésica foi realizada com propofol e a manutenção com isoflurano. As cadelas foram divididas em 2 grupos: em 10 cadelas, foram realizados bloqueios TAP, com um volume de 0,4 mL/kg, associados ao bloqueio serrátil, com um volume de 0,4 mL/kg de bupivacaína a 0,25% intraoperatório, antes de iniciar a cirurgia, e em outras 8 cadelas, a anestesia foi realizada por tumescência com uma solução resfriada composta por Ringer lactato, lidocaína a 2% sem vasoconstritor e adrenalina, em um volume total de 15 mL/kg intraoperatório. Com a coleta de dados, análise, interpretação de resultados e acompanhamento do paciente, foi observado que ambos os grupos não apresentaram estímulos nociceptivos durante o período intraoperatório. E no período pós-operatório, apenas 1 cadela do grupo 1 apresentou uma pontuação maior que 5, e foi observado um leve atraso na recuperação anestésica das cadelas do grupo 2. A avaliação dos parâmetros durante e após a cirurgia, aliada à pouca necessidade de analgésicos na maioria das cadelas, indica que a associação dos bloqueios TAP e SP foi eficaz no controle analgésico. Concluindo que essa combinação se apresenta como uma alternativa interessante para promover analgesia e favorecer a recuperação em cadelas que passarão por mastectomia.

## Introduction

Total unilateral mastectomy is considered invasive and extensive and can result in edema, inflammation, and chronic pain (Gakiya et al., 2011). Postoperative pain from mastectomy procedures in female dogs has been extensively studied, and achieving ideal analgesia in the perioperative period is of great importance. Therefore, a few analgesic protocols have been used for mastectomy (Sarrau et al., 2007). Perioperative analgesia encompasses a combination of preemptive analgesia and locoregional blocks and/or infusions aimed at preventing the development of chronic post-surgical pain and reducing the use of analgesics (Mathews et al., 2014).

The transversus abdominis plane (TAP) block is a locoregional anesthesia technique that inhibits nerve impulse conduction by administering local anesthetics. This approach effectively blocks the genitofemoral nerves and the ventral cutaneous branches of the first three lumbar nerves: cranial iliohypogastric, caudal iliohypogastric, and ilioinguinal, which are responsible for innervating the abdominal and inguinal mammary glands (Campoy et al., 2017a). This new technique is currently being implemented in veterinary medicine using multimodal analgesia protocols. The TAP block has already been used in other studies for mastectomy (Teixeira et al., 2018) to block the abdominal and inguinal mammary glands.

The serratus plane (SP) block is considered an alternative to intercostal nerve blocks, presenting a low risk (Blanco et al., 2013), aiming to provide analgesia to the hemithorax region. Analgesia is achieved through blockade of the lateral cutaneous tissue and branches of the intercostal nerves from T2 to T5, with variable spread potentially reaching T6, rather than through direct blockade of the intercostal nerves (Mayes et al., 2016). The cranial thoracic mammary gland is innervated by lateral cutaneous branches of the 4^th^, 5^th^, and 6^th^ thoracic nerves. The caudal thoracic gland is innervated by the 6^th^ and 7^th^ thoracic nerves and the terminal branches of the ventral cutaneous branches of the 5th and 7th intercostal nerves.

Another technique used for analgesia during mastectomies is tumescent anesthesia, which was first described in 1987 by Klein for liposuction surgery in humans. However, other compositions have been described in the literature (Klein, 1995). Currently, this technique is used in procedures such as liposuction and mastectomy (Vargas et al., 2015). However, limitations to its use exist, such as in cases of poorly delimited tumors or those that are adherent, skin infections, or ulcerated tumors, owing to the risk of post-surgical infection or proliferation of neoplastic cells. A considerable decrease in the patient's temperature intraoperatively occurs due to the administration of the cold solution (Abimussi et al., 2013), which can lead to slower anesthetic recovery.

Mastectomy is a common surgical procedure in clinical practice, and this study aims to evaluate the analgesic potential of combining a block for the abdominal region and another for the thoracic region for pain management in the intraoperative and postoperative periods to improve outcomes by reducing the amount of general anesthetics and analgesics.

## Material and methods

This study was approved by the local Animal Ethics Committee (protocol 016/22). In this study, 18 female dogs aged 5–15 years were selected for mastectomy. The dogs were docile and had no relevant clinical or laboratory findings. All dogs underwent cardiological evaluation with echocardiography and electrocardiography, and only one presented with mitral valve endocardiosis.

The dogs were admitted to the Clínica Escola da Universidade Iguaçu and received 0.03 mg/kg of acepromazine intramuscularly. After 30 min, the cephalic vein was punctured for venous access. Anesthesia was induced with propofol at a rate of 2 mg/kg/min until palpebral and laryngeal reflexes were absent. At this point, the subject was intubated, and administration of isoflurane with 100% oxygen was initiated. Fluid therapy with lactated Ringer's solution was initiated at 5 mL/kg/h.

After this period, the dogs were randomly divided into two groups:

Group 1 (G1): 10 dogs received TAP blocks (Campoy et al., 2017b; Portela et al., 2014; Takeda et al., 2015) at a volume of 0.4 mL/kg, combined with SP blocks at a volume of 0.4 mL/kg of 0.25% bupivacaine intraoperatively, before the start of surgery.

G2: Eight female dogs received tumescent anesthesia (Moraes et al., 2013; Otero et al., 2013; Blanco et al., 2013; Mayes et al., 2016) with a solution composed of 210 mL of lactated Ringer's solution, 40 mL of 2% lidocaine without a vasoconstrictor, and adrenaline (0.5 mL), administered at a rate of 15 mL/kg below the mammary chain.

In case of an acute variation of 20% or more in heart rate or baseline blood pressure, remifentanil was initiated, with total consumption recorded at the end of surgery. The heart rate, respiratory rate, oxygen saturation, and blood pressure were monitored during the anesthetic procedure.

Transoperative analysis was performed by evaluating remifentanil consumption for analgesic management in both groups during mastectomy, as well as assessing post-surgical recovery using the Rioja et al. (2012) scale and pain levels using the Glasgow Scale. If the dog had a score ≥5, they received 0.2 mg/kg of morphine and were reassessed in 30 min. The dog was discharged with a score below 5, at which point they received tramadol (4 mg/kg subcutaneously), meloxicam (0.2 mg/kg subcutaneously), and dipyrone (25 mg/kg subcutaneously). The dogs were sent home with a prescription for tramadol (4 mg/kg orally every 12 h for 5 days), meloxicam (0.2 mg/kg orally once daily for 5 days), and dipyrone (25 mg/kg orally every 8 h for 3 days).

## Results

The 10 female dogs that received the combination of the TAP and SP blocks maintained stable heart rate, respiratory rate, blood pressure, and body temperature throughout the entire intraoperative period, as described in Table 1, with no alteration greater than 20%. Therefore, rescue analgesia with remifentanil was unnecessary during surgery.

**Table 1:** Medições da frequência cardíaca, taxa respiratória, pressão arterial sistólica, média e diastólica, e a medição da temperatura durante o período intraoperatório do grupo que foi submetido ao bloqueio do plano transverso do abdômen (Bloqueio TAP) e ao bloqueio do plano serrátil (Bloqueio SP), em comparação com o grupo que foi submetido ao procedimento de tumescentes.

	*Tap Block + Sp Block*		*Tumescent*
*Heart rate*	98 bpm		94 bpm
*Respiratory frequency*	24 rpm		26 rpm
*Systolic pressure*	95 mmHg		86 mmHg
*Medium pressure*	60 mmHg		60 mmHg
*Diastolic pressure*	48 mmHg		50 mmHg
*Body temperature*	35. 9ºC		35. 1ºC

*Note:* Measurements of heart rate, respiratory rate, systolic, mean and diastolic blood pressure, and temperature measurement throughout the intraoperative period of the group that underwent transversus abdominis plane block (TAP Block) and serratus plane block (SP block) compared to that of the group that underwent tumescent.

However, two of these dogs presented with hypotension during the first blood pressure measurement and before the blocks were performed, requiring the administration of ephedrine (0.03 mg/kg, intramuscular). Shortly afterward, the blood pressure normalized, and a block was performed. In the postoperative period, nine dogs had a pain score below 5 (assessed by the Rioja scale and pain levels by the Glasgow Scale), and they received tramadol, meloxicam, and dipyrone before being discharged. Only one dog had a pain score of 5; therefore, morphine was administered. The dog was reassessed within 30 min, and the pain score was 3; therefore, tramadol, meloxicam, and dipyrone were administered before discharge.

The eight dogs that received tumescent anesthesia maintained stable heart rates, respiratory rates, blood pressures, and body temperatures throughout the entire intraoperative period, as described in Table 1, with no alterations greater than 20%. Therefore, intraoperative rescue analgesia with remifentanil was unnecessary. However, one of these dogs presented with hypotension during the first blood pressure measurement and before the block was performed, requiring the administration of ephedrine (0.03 mg/kg, intramuscular). Shortly afterward, the blood pressure normalized, and a block was performed. In the postoperative period, all eight dogs had pain scores <5 and received tramadol, meloxicam, and dipyrone before discharge. The owners reported that all the dogs in both groups had a smooth postoperative period without apparent pain. The stitches were removed without complications, and all dogs healed well.

The mean vital parameters during surgery are provided in Table 1.

## Discussion

Both groups exhibited no nociceptive stimuli during the intraoperative period, indicating that they did not have alterations in parameters (heart rate, respiratory rate, blood pressure, and body temperature). During the postoperative period, only one dog in G1 had a pain score of 5, which may be attributed to anatomical differences and/or technique failure; however, further studies are necessary to confirm this. All dogs were discharged to their respective homes within 2 h, and 15 days after surgery, the stitches were removed without complications. Contact with the owners was maintained until the stitches were removed, and none of the dogs reported any complications or postoperative pain.

Therefore, both groups had good postoperative outcomes. However, a slight delay in the anesthetic recovery was observed in the dogs in G2, which is believed to be due to their slightly lower body temperatures compared to those in G1. This occurrence can be explained by Abimussi et al. (2013), who explained that when the tumescent technique is used, there may be a considerable decrease in the patient's intraoperative temperature due to the infiltration of the cold solution, even with intraoperative warming strategies. This results in slower anesthetic recovery, as hypothermia leads to consequences such as delayed anesthetic recovery and increased unconsciousness time.

For preanesthetic medication, a protocol that did not provide analgesia was chosen because the objective was to evaluate the analgesic efficacy of the blocks. Acepromazine is a phenothiazine that promotes tranquility and muscle relaxation and has antiemetic and antihistamine effects (Costa et al., 2013). However, it blocks alpha-adrenergic receptors, resulting in peripheral and splenic vasodilation, decreased blood pressure, and reduced hematocrit levels (Tavares et al., 2014). In the current study, two dogs from G1 and one from G2 had slightly increased albumin levels (average 3.6 g/dL). Alterations in the albumin concentration can affect the pharmacokinetics and pharmacodynamics of protein-bound drugs (Conner, 2017). According to Tavares et al. (2014) and Conner (2017), the hypotension observed during the initial measurement, before nerve blocks are performed, can be attributed to the factors described in their study. The female dogs that presented with hypotension did not have cardiac alterations on cardiological evaluation. Ephedrine (0.03 mg/kg, intravenous) administration was necessary.

Teixeira et al. (2018) used a combination of the TAP and SP blocks in dogs undergoing mastectomy. In the SP block, a dorsoventral approach was used to inject 0.3 mL/kg of 0.25% bupivacaine at each of the two points, in the fourth and ninth intercostal spaces, between the thoracic ventral serratus and intercostal muscles. However, because of the absence of anatomical studies on this type of block, the small number of animals used (four), and the chosen site for anesthetic administration (one of the injections was performed in the ninth intercostal space, where the thoracic ventral serratus no longer exists), their study was not conducted under ideal conditions, requiring further research on the subject. Considering this, in the dogs in G1 in this study, the association of blocks was performed in 10 dogs, that is, in a larger number of dogs, and the SP block was performed at two points between the 2^nd^ and 6^th^ intercostal spaces, where it is known that the serratus muscle exists, and none of them presented nociception in the mammary region of the hemithorax.

Teixeira et al. (2018) assessed the combined use of TAP and SP blocks in female dogs undergoing unilateral mastectomy. None of the animals required intraoperative rescue analgesia. Post-extubation, all dogs received prophylactic tramadol (4 mg/kg) and dipyrone (25 mg/kg) as per protocol, and recorded a pain score of 0 at both two and four hours postoperatively.

In a case report by Oliveira et al. (2022), a combination of both techniques was performed, with deep serratus being performed under the fourth and fifth ribs bilaterally, using 0.25% bupivacaine. The TAP block was performed as described by Teixeira (2018) with a single application of 0.25% bupivacaine. In the current study, the same combination of blocks (TAP and SP blocks) was performed with 0.25% bupivacaine, but TAP application was performed at two unilateral points (caudal to the last rib and cranial to the iliac crest), and the serratus was performed unilaterally at two points, thus seeking better drug dissociation.

Sites et al. (2004) reported several disadvantages of ultrasound use, such as difficulty in visualizing the involved structures and failure in needle recognition and localization. In this study, the disadvantages of this technique were not evident, as it was possible to correctly identify the fasciae and position the needle accurately for the injection of the local anesthetic. This may explain why one dog from G1 presented a pain score of 5 in the postoperative period because a partial block exists; that is, analgesia is observed during the intraoperative period, not requiring rescue analgesia, because the dog is anesthetized, and general anesthetics help inhibit pain perception; however, when the patient wakes up, they may experience nociception, as they lose this additional effect of the general anesthetic.

The combination of TAP and SP blocks can replace epidural anesthesia when the technique is contraindicated in patients with neurological deficits, coagulopathies, sepsis, dermatitis at the puncture site, or polytrauma (Klaumann & Otero, 2013; Wetmore & Glowski, 2000). In cases where suppression of potential complications from epidural anesthesia, such as total spinal anesthesia, subdural injection, epidural hematoma, epidural abscess, hypotension, bradycardia, and respiratory depression, is desirable (Cota & Klaumann, 2020). This substitution can improve the outcomes of surgical and anesthetic procedures, thereby reducing the amount of general anesthetic and analgesic required (Campoy et al., 2017b).

In humans, the TAP and SP blocks have been extensively studied. Since its introduction, the SP block has been described and used in surgical procedures involving incisions on the anterolateral thoracic wall, such as mastectomy. The TAP block is used for surgical procedures in the lower abdomen, such as inguinal hernia correction. However, they remain poorly documented in veterinary medicine because they are relatively recent. Several studies have described this technique in dogs undergoing mastectomy, including those by Teixeira et al. (2018) and Oliveira et al. (2022). However, as mentioned earlier, all of these methods have limitations. This study addresses the limitations of previous studies and provides additional information and results that can be incorporated into the existing knowledge.

## Conclusions

Based on the analysis of perioperative parameters and the lack of need for rescue analgesia during the intra- and postoperative periods in most dogs, we observed that the combination of TAP and SP blocks was efficient from an analgesic standpoint. This suggests a good option for alternative blocks to reduce pain and provide better recovery in dogs undergoing mastectomy.
